# Hypothyroidism and Its Rapid Correction Alter Cardiac Remodeling

**DOI:** 10.1371/journal.pone.0109753

**Published:** 2014-10-15

**Authors:** Georges Hajje, Youakim Saliba, Tarek Itani, Majed Moubarak, Georges Aftimos, Nassim Farès

**Affiliations:** 1 Laboratoire de Recherche en Physiologie et Physiopathologie, Faculté de Médecine, Pôle Technologie Santé, Université Saint Joseph, Beirut, Lebanon; 2 Institut National de Pathologie, Baabda, Lebanon; Texas A& M University Health Science Center, United States of America

## Abstract

The cardiovascular effects of mild and overt thyroid disease include a vast array of pathological changes. As well, thyroid replacement therapy has been suggested for preserving cardiac function. However, the influence of thyroid hormones on cardiac remodeling has not been thoroughly investigated at the molecular and cellular levels. The purpose of this paper is to study the effect of hypothyroidism and thyroid replacement therapy on cardiac alterations. Thirty Wistar rats were divided into 2 groups: a control (n = 10) group and a group treated with 6-propyl-2-thiouracil (PTU) (n = 20) to induce hypothyroidism. Ten of the 20 rats in the PTU group were then treated with L-thyroxine to quickly re-establish euthyroidism. The serum levels of inflammatory markers, such as C-reactive protein (CRP), tumor necrosis factor alpha (TNF-α), interleukin 6 (IL6) and pro-fibrotic transforming growth factor beta 1 (TGF-β1), were significantly increased in hypothyroid rats; elevations in cardiac stress markers, brain natriuretic peptide (BNP) and cardiac troponin T (cTnT) were also noted. The expressions of cardiac remodeling genes were induced in hypothyroid rats in parallel with the development of fibrosis, and a decline in cardiac function with chamber dilation was measured by echocardiography. Rapidly reversing the hypothyroidism and restoring the euthyroid state improved cardiac function with a decrease in the levels of cardiac remodeling markers. However, this change further increased the levels of inflammatory and fibrotic markers in the plasma and heart and led to myocardial cellular infiltration. In conclusion, we showed that hypothyroidism is related to cardiac function decline, fibrosis and inflammation; most importantly, the rapid correction of hypothyroidism led to cardiac injuries. Our results might offer new insights for the management of hypothyroidism-induced heart disease.

## Introduction

It is estimated that more than 12% of the US population will develop a thyroid condition during their lifetime, and an estimated 20 million Americans have already some form of thyroid disease [1]. Besides, some of the most prominent and common symptoms of thyroid disease are those that result from the effects of thyroid hormone on the heart and cardiovascular system [2], [3], [4], [5], [6], [7], [8], [9], [10], [11]. Both hyperthyroidism and hypothyroidism produce changes in cardiac contractility, myocardial oxygen consumption, cardiac output, blood pressure, and systemic vascular resistance [12], [13], [14], [15], [16], [17].

Several important cardiac structural and functional proteins are transcriptionally regulated by thyroid hormones. The proteins that are positively regulated by thyroid hormones include sarcoplasmic reticulum calcium ATPase (SERCA2) that uptakes calcium into the sarcoplasmic reticulum during diastole [4], [18], alpha myosin heavy chain (α-MHC) the fast myosin with higher ATPase activity as well as beta adrenergic receptors, sodium/potassium ATPase and voltage-gated potassium channels Kv1.5, Kv4.2 and Kv4.3 which together coordinate the electrochemical responses of the myocardium [3], [6], [17], [19], [20], [21], [22], [23], [24], [25]; cardiac stress markers atrial and brain natriuretic peptides (ANP and BNP) are also regulated by thyroid hormones [19], [20]. Other cardiac proteins are negatively regulated by thyroid hormones such as β-MHC the slow myosin, phospholamban the SERCA inhibitor and sodium/calcium exchanger [5], [19], [20], [26], [27]. Thus, changes in the amounts of these proteins account for the altered cardiac diastolic and systolic function induced by thyroid disease.

Non-genomic effects are also exerted by thyroid hormones on cardiac myocytes and ion transport [26], [28]. In fact, triiodothyronine (T3) exerts effects on various sodium, potassium, and calcium channels in the heart, and thus changes in intracellular levels of calcium and potassium can increase inotropy and chronotropy [5], [29], [30].

Hypertrophied and, in particular, failing hearts are characterized by an accumulation of extracellular matrix elements and a corresponding increase in cardiac muscle stiffness [31], [32], [33], [34], [35], [36], [37], [38], [39], [40]. Fibronectin and collagen types I and III are the major components of the interstitial fibrillar network [41], [42], [43], [44]; thus, it has been hypothesized that the up-regulation of fibroblasts genes encoding these components accounts, in part, for the increase in fibrosis observed during the transition to heart failure and contributes to the decline in contractile performance [31], [45], [46], [47], [48], [49], [50]. The elaboration of the extracellular matrix by fibroblasts is influenced by TGF-β1 and plays an important role in pressure-overload cardiac hypertrophy [51], [52], [53]. The cytokine TGF-β1 is expressed by and modulates myocytes, vascular cells and fibroblasts [54], [55]; its expression rises in myocardium in experimental and human heart disease [55], [56], and it promotes hypertrophy, fibrosis, apoptosis, and endothelial-mesenchymal transition [57], [58], [59], [60].

Furthermore, a generalized increase in the level of contractile proteins, such as β-MHC and myofibroblast marker alpha smooth muscle actin (α-SMA), constitutes a marker of cardiac hypertrophy [61], [62]. Shifts from the normally predominant α-MHC toward β-MHC are often observed in cardiomyocytes from hypertrophied and failing hearts [63], [64], [65], [66]. α-SMA is expressed in cardiomyocytes during early stages of heart development and in dedifferentiated cardiac fibroblasts and its reactivation is considered a potential marker of ventricular hypertrophy [67], [68], [69]. Finally, the BNP serum level is also considered to be one of several criteria indicating the initiation of a pathological response in hypertrophied failing hearts [70], [71], [72]. Cardiac and circulating pro-inflammatory markers such as CRP, interleukins and TNF-α have been also associated with cardiac disease [73].

Severe hypothyroidism caused dilated cardiomyopathy with decreased α-MHC and increased β-MHC, phospholamban and ANP, an accepted clinical marker of the diseased hypertrophic heart [74], [75]. After thyroid hormone replacement the alterations in gene expression were reversed with overall improvement in myocardial performance [75]. In keeping, thyroid replacement therapy has been used by many investigators to treat patients with heart failure [76], [77], [78], [79], [80]. However, the beneficial therapeutic effects obtained in the short term might be followed by pathological manifestations of the systemic effects of thyroid hormones during longer treatments [81], [82], [83], [84].

The 6-propyl-2-thiouracil (PTU) drug which is a thiouracil-derivative used to treat hyperthyroidism [85], [86] has been long used to induce hypothyroidism in animal models such as the rat [9], [87], [88]. Data obtained from animal models on the effect of thyroid hormone on myocardial gene expression are further supported by clinical studies on humans [74], [75].

Most studies on the cardiovascular effects of thyroid hormones have particularly targeted left ventricular improvement. Nonetheless, little is known about the effect of an excess or deficiency of thyroid hormones on the myocardium collagen gene and the responsiveness of interstitial cells to these hormones [89], [90], [91]. One study, however, showed that long term hypothyroidism caused overall cardiac muscle stiffness and left ventricle diastolic wall thickness due to increased collagen I/III-based stiffness [92]. Furthermore, to our knowledge, no studies have evaluated the combined effect of hypothyroidism and thyroid hormone therapy on cardiac inflammation. The purpose of this study was to evaluate the cardiac fibrosis, inflammation and dysfunction caused by PTU-induced hypothyroidism in adult rats. Additionally, we examined the reversibility of these changes after the establishment of a euthyroid state.

## Materials and Methods

### Ethical approval

The protocols of the present study were approved by the Ethical Review Committee of the Saint Joseph University, were performed in accordance with the Guiding Principles in the Care and Use of Animals approved by the Council of the American Physiological Society and adhered to the *Guide for the Care and Use of Laboratory Animals* published by the US National Research Council committee.

### Animals and experimental design

Thirty adult male Wistar rats weighing 250±25 g were obtained from the “Centre d′Elevage R. Janvier” (Le Genest-Saint Isle, France). The animals were housed at a stable temperature (25°C) and humidity and were exposed to a 12∶12 h light-dark cycle. The rats were fed ordinary rat chow, had free access to tap water and were acclimatized for at least one week under these conditions before the start of the study. Then, all animals were weighed once per week.

As shown in Figure S1, the animals were divided randomly into 2 groups: one group of 10 rats (control group) and a second group of 20 rats treated with PTU (Sigma-Aldrich, Germany); PTU was added to the drinking water for 6 weeks at a final concentration of 0.1 g/100 ml, as previously described [9], [93], [94], [95], [96], [97], [98], [99], [100]. In fact, PTU inhibits iodine and the thyroperoxidase enzyme from their normal interactions with the hormone precursor thyroglobulin to form T4 and T3. This action decreases production of thyroid hormone. In addition to these well recognized effects on intra-thyroidal iodine metabolism, PTU inhibits the peripheral deiodination and conversion of T4 into the more potent form T3 by inhibiting the 5'-deiodinase enzyme [101], [102], [103], [104]. At the end of the 6 weeks, 10 rats from the PTU group were treated with a solution of 6 µg/ml of Euthyrox (L-thyroxine-LT4, Merck, Germany), which was added to the drinking water for one week to quickly reverse the hypothyroidism (reverse group) and recover the euthyroid state; these rats were then sacrificed five weeks later. This L-thyroxine dose was chosen as previously described to maximize left ventricular function and improve heart remodeling without the risk of possible thyrotoxicosis [105], [106], [107], [108]. The rats of the control group and the remaining PTU-treated rats (n = 10) that were not treated with LT4 were sacrificed at the same time as the reverse group. Prior to sacrifice, echocardiography and blood analysis (see below), all animals were anesthetized with a mixture of ketamine (Interchemie, Holland, 75 mg/kg) and xylazine (Rotex Medica, Germany, 10 mg/kg) and were completely non-responsiveness to toe pinching.

After the sacrifice, the heart of the rats was excised, dry-weighted then cut in half; one half was kept in a 4% formalin solution (Sigma-Aldrich, St. Louis, Missouri, USA) for histopathology assessment, and the other half was stored at −80°C for the eventual RNA extraction.

### Blood analysis

The levels of free triiodothyronine (fT3), free thyroxine (fT4), thyroid-stimulating hormone (TSH), BNP, CRP, IL6, TNF-α, TGF-β1, cTnT, adiponectin and leptin were measured in the serum of all the rats. Three blood samples (0.5 ml) were collected from each anesthetized rat at three distinct times: after 6 weeks of PTU treatment, after the 1 week of LT4 treatment to check for the rapid establishment of the euthyroid state and at the time of sacrifice (Figure S1).

Blood samples were collected in EDTA tubes via the jugular vein. The blood samples were immediately centrifuged at 3500 rpm for 10 min, and the serum was frozen at −80°C for subsequent analyses. The BNP, CRP, IL6, TNF-α, TGF-β1, adiponectin and leptin ELISA kit reagents were purchased from Abcam, Cambridge - UK, and the fT3, fT4, TSH and cTnT ELISA kit reagents were purchased from CusaBio, Hubei, China. All samples were run in triplicate, and the intra- and inter-assay variations were less than 10%.

### Echocardiographic assessment

The animals were anesthetized by 1.5% isoflurane gas and placed on isothermal pads with their chests shaved. Trans-thoracic two-dimensional guided M-mode echocardiography was performed via the long axis of the left ventricle at the level of the papillary muscles using a SonoScape instrument equipped with a convex 8 MHz transducer. The surgeon and echocardiographer were blinded to the rats' pre-treatments.

### Histopathology

The formalin-fixed heart tissues were embedded in paraffin, and 4-µm thick sections were cut. Paraffin-embedded sections of the hearts were stained with either hematoxylin-eosin or Masson's trichrome (Sigma-Aldrich, St. Louis, Missouri, USA) for histopathological evaluation. After staining, the sections were dehydrated in ethanol/water baths with decreasing water content and finally rinsed in xylene before being mounted with a permanent mounting medium.

Gross examination and histological sections were analyzed by two independent pathologists in a blinded fashion. A semi-quantitative scoring system was used to assess the cardiac myocyte hypertrophy, tissue inflammation and fibrosis. Inflammation refers to the presence of aggregates of leukocytes in the heart muscle. Fibrosis analysis was performed using the ImageJ program by applying a threshold to the acquired pictures and creating selections of the fibrotic areas. Eight sections were analyzed for each rat in the three groups.

### Real-time quantitative polymerase chain reaction

Hearts previously kept at −80°C were ground, and total cellular RNA was extracted from cells using TRIzol reagent (Life Technologies, Carlsbad, California, USA). The samples were then purified with 75% ethanol, and the purity and concentration of the RNA were determined by measuring the absorbance at 260 nm with an ND-1000 NanoDrop spectrophotometer (Thermo Scientific, Wilmington, Delaware, USA). Subsequently, the first-strand cDNA was synthesized using a Superscript III kit for RT-PCR (Life Technologies, Carlsbad, California, USA). Briefly, total RNA was denatured at 65°C for 5 min in the presence of 250 ng/µl random primers and 10 mM deoxynucleotide triphosphate. After the sample was chilled on ice for at least 1 min, reverse transcription was allowed to proceed at 25°C for 5 min in the presence of 1× first-strand buffer, 5 mM dithiothreitol, and 40 U of ribonuclease inhibitor. The reaction was then allowed to proceed at 50°C for another 60 min. The reaction was terminated by heat inactivation at 70°C for 10 min. Real-time PCR was conducted using a 7500 real-time PCR system (Applied Biosystems, USA) and SYBR Green PCR master mix (Life Technologies, Carlsbad, California, USA). Real-time Q-RT-PCR was performed in a 20-µl reaction mixture containing 250 nM forward and reverse primers, 1× SYBR Green reaction mix and various amounts of template. The reaction was performed with preliminary denaturation for 10 min at 95°C to activate Taq DNA polymerase, followed by 40 cycles of denaturation at 95°C for 15 sec and annealing/extension at 60°C for 1 min. The fluorescence emitted by SYBR Green was detected online by the ABI PRISM 7500 sequence detection system (Applied Biosystems). Melting curves were measured at the end of the amplification to confirm the specificity of the amplified products. All PCR was performed in triplicate on the same 96-well plate. Ribosomal protein L32 (Rpl32) was used as a housekeeping gene, and quantifications were conducted using the 2^ΔΔCt^ method. Studies have shown that the initial copy number can be quantitated during real-time PCR analysis based on the threshold cycle (Ct) [109], [110]. The Ct is defined as the cycle at which fluorescence is determined to be significantly greater than the background. For quantification of the gene expression changes, the Ct method was used to calculate the relative fold changes normalized against the Rpl32 gene. To check for genomic DNA contamination, parallel samples were run without the addition of the reverse transcriptase. The primers (Eurogentec, Seraing, Belgium) used are presented in Table 1.

**Table 1:** Primers for the different genes.

Gene	Forward primer (5′-3′)	Reverse primer (5′-3′)
***Anp***	CTTCCTCTTCCTGGCCTTTTG	CGCACTGTATACGGGATTTGC
***Bnp***	AAGTCCTAGCCAGTCTCCAGAACA	AGCTCCAGCAGCTTCTGCAT
***α-Mhc***	CTTCTGCTGATACCGGTGACAG	TGAGCCTTTCTTCTTGCCTCC
***β-Mhc***	ACAGGAAAGTTGGCATCTGCA	GGATTTTTCCAGAAGGTAGGTCTCT
***α-Sma***	AGAGTGGAGAAGCCCAGCCAGTC	ATCATCACCAGCAAAGCCCGCC
***Tgf-β1***	ATGGTGGACCGCAACAACGCAATC	CACGGGACAGCAATGGGGGTT
***cTgf***	TGTGCACGGAGCGTGATCCC	TGCACCATCTTTGGCAGTGCACA
***Il1***	CTGACAGACCCCAAAAGATTAAGG	CCTTGTCGAGATGCTGCTGTGA
***Mcp1***	CAGCCAGATGCAGTTAATGCCCC	GCTTCTTTGGGACACCTGCTGCTG
***Col1***	GAGAGAGCATGACCGATGGATT	GATAGCGACATCGGCAGGAT
***Col3***	AAACGGAGAACGGGGTGGCC	TCACCAGGTGCGCCAGTAGGT
***Rpl32***	CCAGAGGCATCGACAACA	GCACTTCCAGCTCCTTGACAT

Forward and reverse primers used for quantitative real-time PCR. Abbreviations: *Anp*: Atrial natriuretic peptide; *Bnp*: Brain natriuretic peptide; *α-Mhc*: α-Myosin heavy chain; *β-Mhc*: β-Myosin heavy chain; *α-Sma*: α-Smooth muscle actin; *Tgf-β1*: Transforming growth factor beta 1; *cTgf*: Connective tissue growth factor; *Il1*: Interleukin 1; *Mcp1*: Monocyte chemoattractant protein 1; *Col1*: Collagen type I; *Col3*: Collagen type III; *Rpl32*: Ribosomal protein L32.

### Statistical analysis

All quantitative data are reported as the mean ± SEM. Statistical analysis was performed with the SigmaPlot (v11.0) software. Kruskal-Wallis One-way ANOVA on rank tests were performed for multiple comparisons of the values because the normal distribution verified with the Shapiro-Wilk test was not met. Post hoc analysis was performed with the Dunn's multiple comparison tests to identify the group differences that accounted for the overall ANOVA results. When two independent variables were present, two-way ANOVA tests were performed, followed by post hoc Tukey's multiple comparison tests. All values with *p*<0.05 were considered significant.

## Results

### Serum levels of fT3, fT4 and TSH

**Figure 1:**
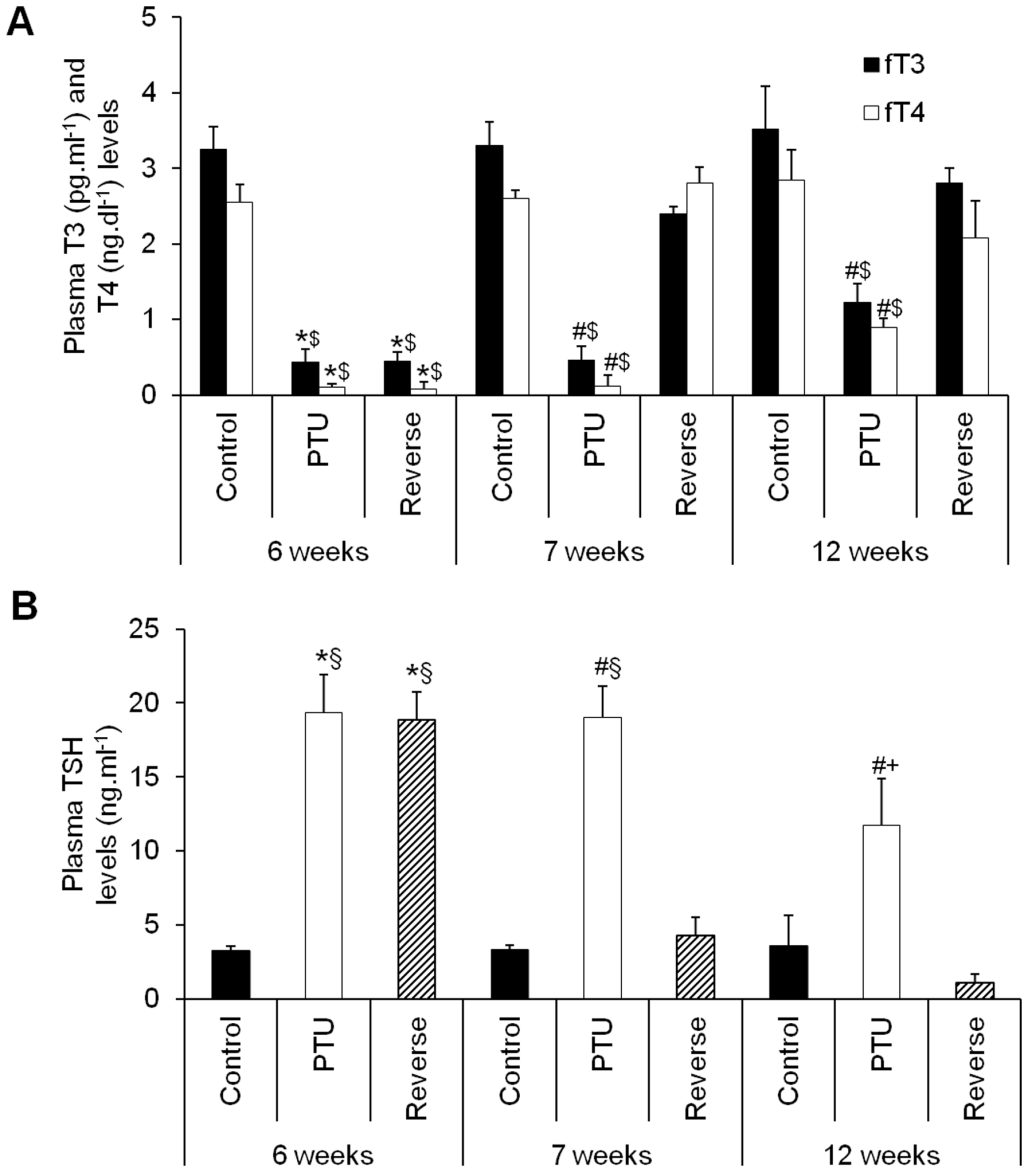
Serum levels of fT3 (A), fT4 (A) and TSH (B) in the different rat groups at different times after the PTU or LT4 treatments and sacrifice. Data are represented as the mean ± SEM. n = 10 animals for each group. Statistical analysis was performed with two-way ANOVA tests followed by post hoc Tukey's multiple comparison tests. *p<0.001 *vs.* control; ^#^ *p*<0.001 *vs.* control and reverse; ^$^ *p*<0.001 *vs*. 7 and 12 weeks reverse and 12 weeks PTU; ^§^ *p*<0.001 *vs.* 7 and 12 weeks reverse; ^+^ *p*<0.001 *vs*. 7 and 12 weeks reverse and 7 weeks PTU.

Administration of PTU for 6 weeks resulted in significant decreases in fT3 and fT4 compared with the levels in the control rat group, confirming the establishment of hypothyroidism. Treatment with LT4 for 1 week raised fT3 and fT4 to levels comparable to those of the control rats, verifying the rapid reversibility of hypothyroidism (Figure 1A). After 12 weeks, at the time of sacrifice, the PTU group still presented relatively low levels of fT3 and fT4, whereas the reverse group retained levels comparable to those of the control rats (Figure 1A).

The TSH levels significantly increased under the PTU treatment, which also confirmed the hypothyroid state, and remained elevated at 12 weeks even with the discontinuation of PTU (Figure 1B). The LT4 treatment for 1 week decreased the TSH levels to normal values that remained stable until the end of the protocol (Figure 1B).

### Plasma markers of inflammation, fibrosis and cardiac hypertrophy

**Figure 2:**
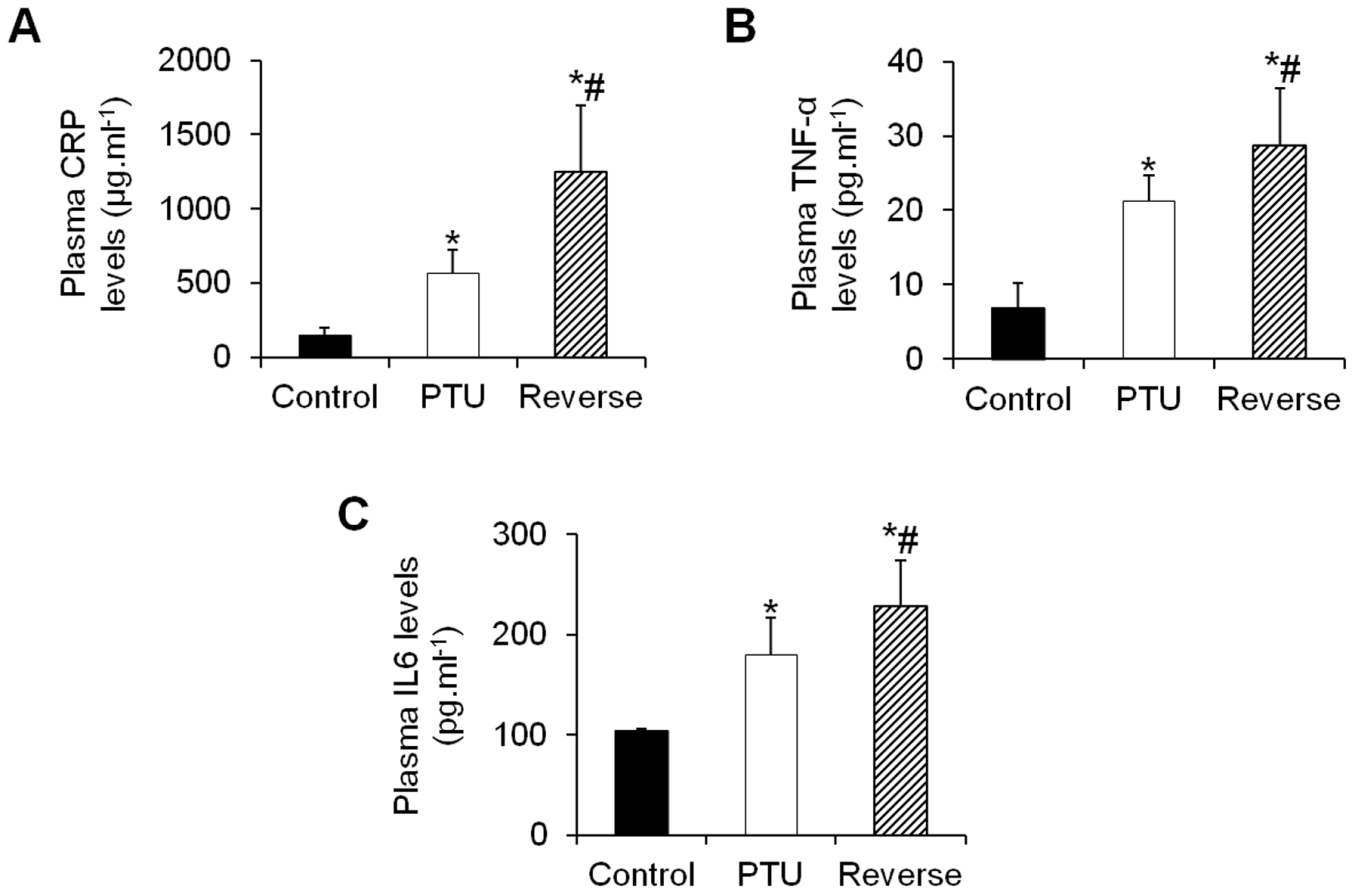
Effects of hypothyroidism and LT4 treatment on the plasma levels of inflammatory markers. Changes in the CRP (**A**), TNF-α (**B**) and IL6 (**C**) levels in the different treatment groups at the time of sacrifice. Data are represented as the mean ± SEM. n = 10 animals for each group. Statistical analysis was performed with Kruskal-Wallis One-way ANOVA on rank tests followed by post hoc Dunn's multiple comparison tests. *p<0.01 *vs.* control; ^#^ *p*<0.05 *vs.* PTU.

**Figure 3:**
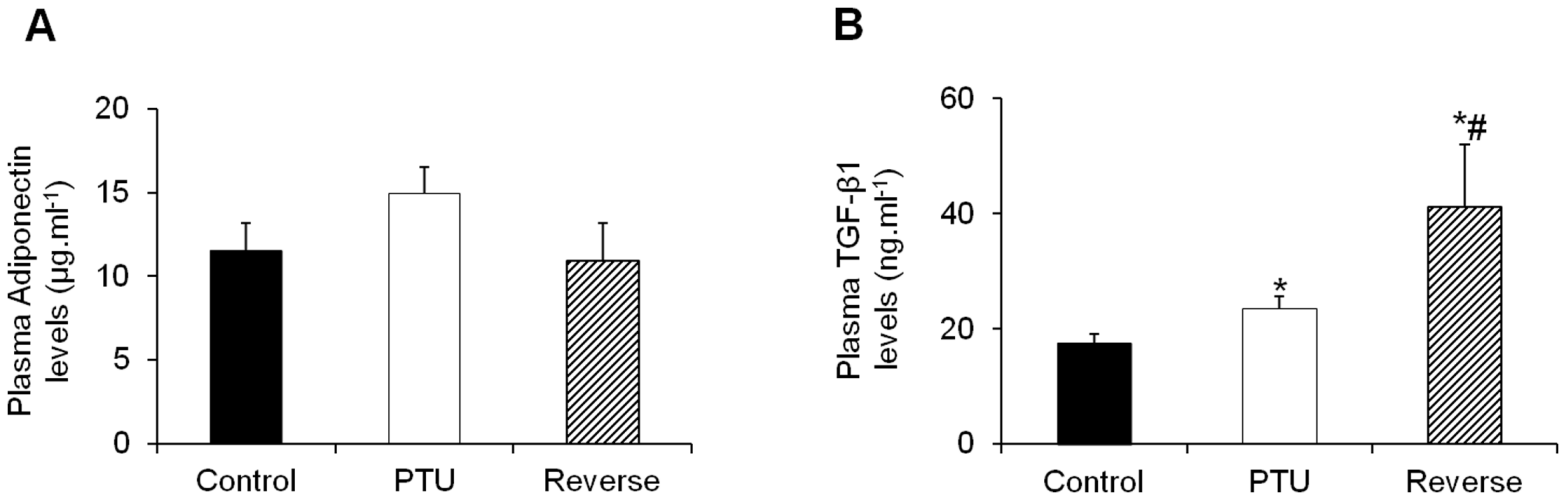
Adiponectin (A) and TGF-β1 (B) plasma levels in the different groups of animals. Data are represented as the mean ± SEM. n = 10 animals for each group. Statistical analysis was performed with Kruskal-Wallis One-way ANOVA on rank tests followed by post hoc Dunn's multiple comparison tests. *p<0.01 *vs.* control; ^#^ *p*<0.05 *vs.* PTU.

**Figure 4:**
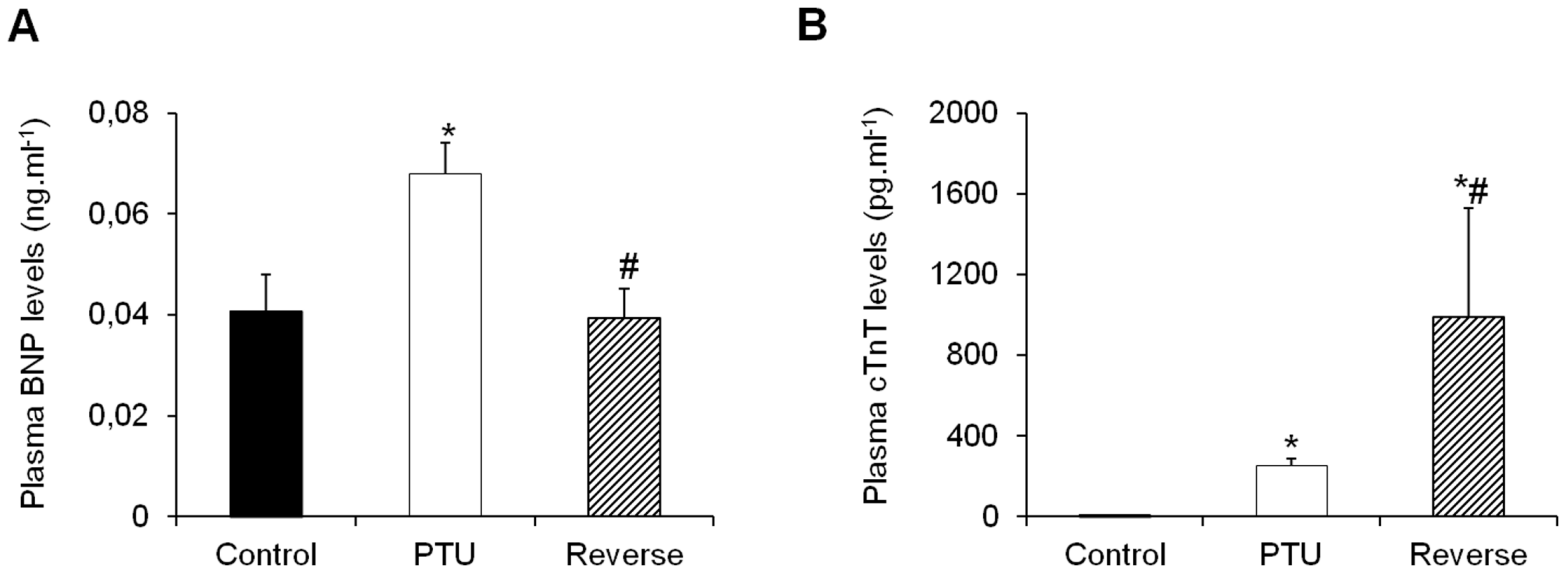
Effects of hypothyroidism and LT4 treatment on the plasma levels of cardiac stress markers. Changes in the BNP (**A**) and cTnT (**B**) levels in the different groups. Data are represented as the mean ± SEM. n = 10 animals for each group. Statistical analysis was performed with Kruskal-Wallis One-way ANOVA on rank tests followed by post hoc Dunn's multiple comparison tests. *p<0.05 *vs.* control; ^#^ *p*<0.01 *vs.* PTU.

Hypothyroidism resulted in significant increases in the plasma inflammatory markers CRP and TNF-α and IL6, whereas reversing the hypothyroid state further increased these levels (Figure 2A–2C). The level of adiponectin, a cardioprotective adipocyte-derived cytokine, did increase in the PTU-treated rats; however, this increase was not significant. The mean plasma levels of adiponectin returned to normal after the LT4 treatment (Figure 3A). The mean plasma level of pro-fibrotic TGF-β1 increased in the PTU rats and drastically increased after the hypothyroidism reversal (Figure 3B). Similarly, there was a significant increase in the plasma cardiac stress marker BNP in the PTU group, and this level returned to normal under the LT4 treatment (Figure 4A). In addition, the cTnT levels dramatically increased in the PTU group compared with the levels in the control group and were further induced after the hypothyroidism reversal (Figure 4B).

### Gene expression of cardiac remodeling markers

**Figure 5:**
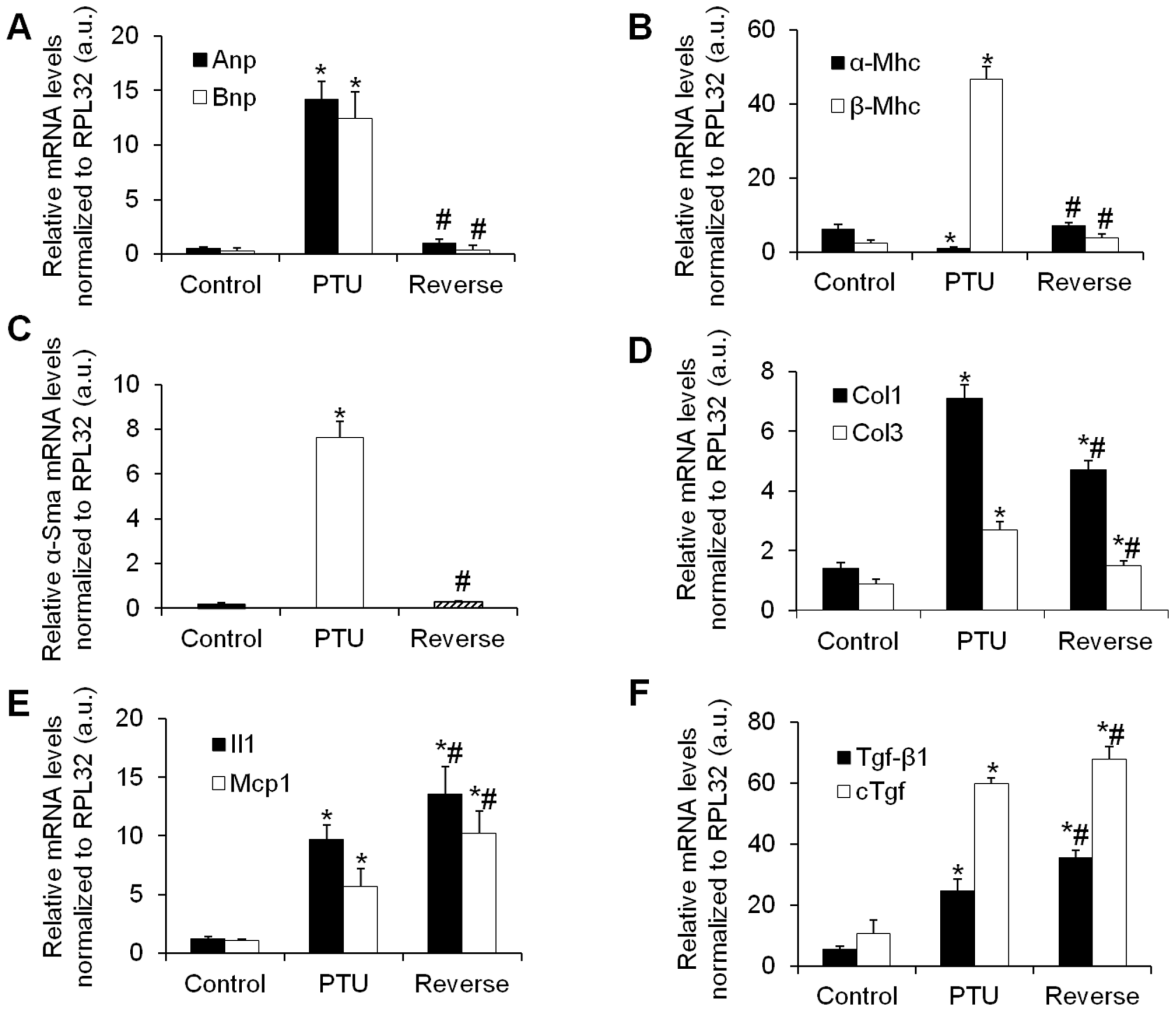
Effects of hypothyroidism and LT4 treatment on hypertrophic, fibrotic and inflammatory gene markers in the rat heart. *Anp*, *Bnp* (**A**), *α-Mhc*, *β-Mhc* (**B**), *α-Sma* (**C**), *Tgf-β1*, *cTgf* (**D**), *Il1*, *Mcp1* (**E**) and *Col1*, *Col3* (**F**) gene expressions relative to the housekeeping gene *Rpl32*. Data are represented as the mean ± SEM. n = 10 animals for each group. Statistical analysis was performed with Kruskal-Wallis One-way ANOVA on rank tests followed by post hoc Dunn's multiple comparison tests. a.u.: arbitrary units. *p<0.01 *vs.* control; ^#^ *p*<0.01 *vs.* PTU.

Hypothyroidism induced the expression of the fetal genes for atrial and brain natriuretic peptides (*Anp* and *Bnp*, respectively) in the heart, whereas LT4 treatment abolished these expressions (Figure 5A). The expression of the cardiac muscle-specific protein *α-Mhc*, which is involved in active force generation, decreased in the hypothyroid rats, whereas the LT4 treatment returned this gene expression to levels comparable to those in the control rats (Figure 5B). In contrast, the slow-twitch *β-Mhc* isoform expression drastically increased in the hypothyroid rats and then returned to normal under LT4 treatment (Figure 5B).

The cardiac fibroblast to myofibroblast differentiation, as shown by *α-Sma* expression, increased in the hypothyroid rats and then returned to normal after the euthyroid state was established (Figure 5C), whereas the cardiac extracellular matrix components *Col1* and *Col3* presented the same gene expression profile (Figure 5D).

Interestingly, the pro-fibrotic markers *Tgf-β1* and *cTgf* and the inflammatory markers *Il1* and *Mcp1* presented expression profiles that were opposite to those of the other cardiac stress markers. In fact, *Tgf-β1* and *cTgf* and *Il1* and *Mcp1* were induced by PTU and further increased under LT4 treatment (Figure 5E and 5F).

### Echocardiography and cardiac hypertrophy assessment

**Figure 6:**
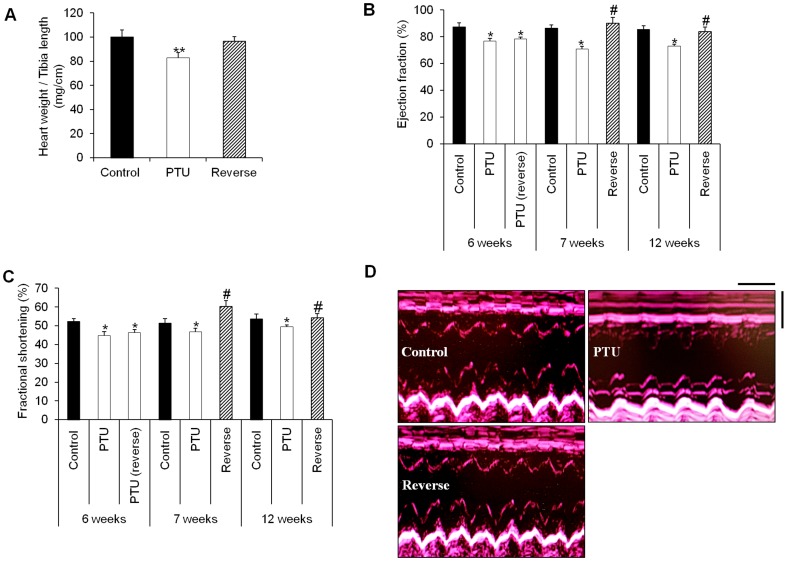
The LT4 treatment reversed cardiac dilation and improved systolic function. **A**: Heart weight to tibia length ratio (mg/cm) in the different treatment groups. Statistical analysis was performed with Kruskal-Wallis One-way ANOVA on rank tests followed by post hoc Dunn's multiple comparison tests. **p<0.01 *vs.* control and reverse. **B** and **C**: Echocardiographic measurements of the ejection fraction (%) and fractional shortening (%) in the control, PTU-treated and LT4-treated rats at different time points. Statistical analysis was performed with two-way ANOVA tests followed by post hoc Tukey's multiple comparison tests. *p<0.05 *vs.* control as well as 7 and 12 weeks reverse; ^#^ *p*<0.05 *vs.* PTU. **D**: M-mode echocardiographic tracings from the control, PTU and reverse groups. Echocardiography scale bars: 0.25 s and 0.5 mm. Data are represented as the mean ± SEM. n = 10 animals for each group.

Significant decreases in heart mass were present during the PTU treatment (Table 2) along with a reduction of the ratio of the heart weight to tibia length (Figure 6A). This heart weight loss was abolished by the LT4 treatment (Table 2, Figure 6A). In addition, the growth rate was significantly inhibited in the rats treated with PTU compared with that in the control group (Table 2), although the PTU-treated animals appeared to be reasonably healthy, with no changes in water intake and food consumption. This effect was observed very early after the beginning of the treatment. Furthermore, it was reversed after the re-establishment of a euthyroid state. At the end of the study, there was no significant difference in the mean body weight between the reverse group and the control group (Table 2). Concordantly, the “satiety hormone” leptin showed significant plasma elevations under the PTU treatment, and these elevations regressed to normal levels after hypothyroidism reversal (Figure S2). This reduction in body mass with PTU treatment affected the heart weight to body weight ratio of the rats in an opposite way as compared to the heart weight to tibia length ratio (Table 2).

**Table 2:** Body and heart weights in the different rat groups.

Groups	n	Body weight (BW), g	Heart weight (HW), mg	HW/BW (mg/g)
Control	10	404 ± 17.6	1582.8 ± 124	3.92 ± 0.3
PTU	10	248 ± 37.6*	1212.4 ± 95*	4.88 ± 0.38*
Reverse	10	406 ± 19.2^#^	1566.6 ± 156^#^	3.86 ± 0.38^#^

Data are represented as the mean ± SEM. Statistical analysis was performed with Kruskal-Wallis One-way ANOVA on rank tests followed by post hoc Dunn's multiple comparison tests. n: number of animals in each group. *p<0.001 *vs.* control; ^#^ *p*<0.001 *vs.* PTU.

Echocardiography showed significant bradycardia after the hypothyroidism induction at 6 weeks, and these decreases in heart rate were still noticeable at 12 weeks (Table 3). In the PTU group, the end-diastolic and end-systolic interventricular septum thicknesses (IVSTd and IVSTs, respectively) were significantly reduced compared with those in the control animals, whereas the end-diastolic and end-systolic left ventricular diameters (LVIDd and LVIDs, respectively) increased, indicating the presence of left ventricular dilation. Similarly, the end-diastolic and end-systolic posterior wall thicknesses (LVPWd and LVPWs, respectively) showed significant regressions in the PTU group (Table 3). All of these parameters were reversed under the LT4 treatment, demonstrating the reversal of cardiac dilation (Table 3).

**Table 3:** Echocardiographic parameters of the rats at different time periods after hypothyroidism induction and LT4 treatment.

		HR (bpm)	IVSTd (cm)	LVIDd (cm)	LVPWd (cm)	IVSTs (cm)	LVIDs (cm)	LVPWs (cm)
**6 weeks**	**Control**	475 ± 12	0.14 ± 0.02	0.65 ± 0.05	0.13 ± 0.02	0.22 ± 0.02	0.31 ± 0.06	0.21 ± 0.02
	**PTU**	335 ± 10*	0.11 ± 0.01*	0.87 ± 0.07*	0.11 ± 0.02*	0.18 ± 0.03*	0.48 ± 0.05*	0.18 ± 0.01*
	**PTU (reverse)**	327 ± 14*	0.1 ± 0.02*	0.84 ± 0.09*	0.11 ± 0.01*	0.19 ± 0.03*	0.45 ± 0.07*	0.17 ± 0.02*
**7 weeks**	**Control**	490 ± 18	0.15 ± 0.02	0.68 ± 0.06	0.14 ± 0.02	0.23 ± 0.03	0.33 ± 0.06	0.21 ± 0.02
	**PTU**	411 ± 11*	0.12 ± 0.02*	0.92 ± 0.1*	0.12 ± 0.01*	0.20 ± 0.02*	0.49 ± 0.05*	0.18 ± 0.01*
	**Reverse**	500 ± 17^#^	0.15 ± 0.03^#^	0.78 ± 0.08^#^	0.16 ± 0.02^#^	0.22 ± 0.02^#^	0.31 ± 0.07^#^	0.23 ± 0.01^#^
**12 weeks**	**Control**	480 ± 20	0.16 ± 0.02	0.67 ± 0.11	0.16 ± 0.01	0.25 ± 0.03	0.31 ± 0.04	0.23 ± 0.02
	**PTU**	410 ± 15*	0.13 ±0.01*	0.85 ± 0.05*	0.14 ± 0.01*	0.21 ± 0.02*	0.43 ± 0.03*	0.19 ± 0.02*
	**Reverse**	465 ± 22^#^	0.15 ±0.01^#^	0.72 ± 0.05^#^	0.17 ± 0.02^#^	0.26 ± 0.02^#^	0.33 ± 0.05^#^	0.22 ± 0.01^#^

HR: heart rate; IVSTd: end-diastolic interventricular septum thickness; LVIDd: end-diastolic left ventricular diameter; LVPWd: end-diastolic posterior wall thickness; IVSTs: end-systolic interventricular septum thickness; LVIDs: end-systolic left ventricular diameter; LVPWs: end-systolic posterior wall thickness. Data are represented as the mean ± SEM. Statistical analysis was performed with two-way ANOVA tests followed by post hoc Tukey's multiple comparison tests. n = 10 rats in each group. *p<0.05 *vs.* control as well as 7 and 12 weeks reverse; ^#^ *p*<0.05 *vs.* PTU.

The cardiac function declined after the PTU treatment, and the ejection fraction and fractional shortening values decreased (Figure 6B and 6C). These changes were quickly reversed after just the 1 week LT4 treatment and remained unchanged until 12 weeks (Figure 6B and 6C). Representative examples of M-mode echocardiography tracings from the different groups are shown in Figure 6D.

### Histopathology

**Figure 7:**
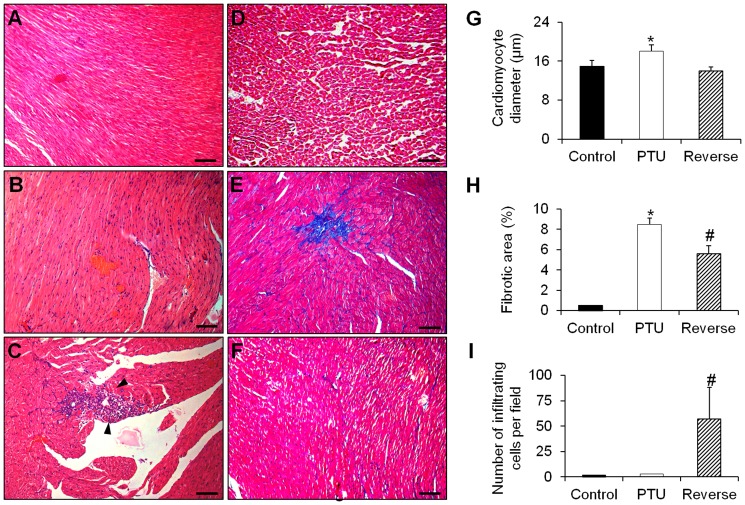
Representative microphotographs of histological slices of hearts from the different rat groups. **A** and **D**: Heart of the control group stained with hematoxylin-eosin (**A**) and Masson's trichrome (**D**) showing a well-organized lamellar arrangement of cardiomyocytes without signs of fibrosis. **B** and **E**: Heart of the PTU group stained with hematoxylin-eosin (**B**) and Masson's trichrome (**E**) showing cardiomyocyte hypertrophy with increased size of the nuclei and presenting zones of fibrosis (blue in **E**). **C** and **F**: Heart of the reverse group stained with hematoxylin-eosin (**C**) and Masson's trichrome (**F**) showing a massive infiltration of lymphocytes and macrophages (arrows in **C**) and diminished fibrotic lesions (blue in **F**). **G–I**: Quantitative analysis of cardiomyocyte size, fibrotic areas and cellular infiltration in each of the different animal groups. Fibrosis analysis was performed using the ImageJ program by thresholding the acquired pictures and then creating selections of the fibrotic areas. Scale bars: 75 µm. Data are represented as the mean ± SEM. n = 10 animals for each group. Eight sections were analyzed for each rat in the three groups. Statistical analysis was performed with Kruskal-Wallis One-way ANOVA on rank tests followed by post hoc Dunn's multiple comparison tests. *p<0.01 *vs.* control and reverse; ^#^ *p*<0.01 *vs.* control and PTU.

Hypothyroidism resulted in a significant increase in the nucleus size of the myocardial cells as well as the development of myocardial hypertrophy (Figure 7A, 7B and 7G) and focal fibrosis lesions (Figure 7D, 7E and 7H), compared with the control group. There was no inflammatory cell infiltration and no signs of myocardial lesions or myocarditis (Figure 7A, 7B and 7I).

However, in the reverse group, the myocardial hypertrophy and fibrosis regressed (Figure 7C and 7F-7H). Most importantly, there was massive infiltration of inflammatory cells more specifically macrophages and lymphocytes, whereas signs of myocardial lesions and myocarditis were clearly developed (Figure 7C and 7I).

## Discussion

Our study showed that hypothyroidism in rats is related to the development of cardiac fibrosis and inflammation along with chamber dilation and function decline. Interestingly, the rapid re-establishment of euthyroidism resulted in aggravated cardiac inflammation and injury, although the fibrosis and function were contradictorily ameliorated. Thyroid replacement therapy in hypothyroid patients has been shown to improve their prognosis and reduce their cardiovascular risk [5], [108], [111], [112], [113], [114], [115]. However, some hypothyroid patients might remain at an increased risk for the morbidity associated with circulatory diseases and ischemic heart disease as well as other systemic manifestations despite treatment with LT4 [81], [116], [117], [118], [119].

After 6 weeks of PTU treatment, the fT3 and fT4 levels were depressed, whereas the TSH level drastically increased, indicating the establishment of hypothyroidism. The normal levels of fT3 and fT4 following the one week treatment with LT4 confirmed the rapid reversal of hypothyroidism. Intriguingly, the levels of fT3 and fT4 rose but still remained significantly low with high TSH 6 weeks after the PTU treatment was stopped in the animals that did not receive LT4. This apparent discrepancy in the levels of thyroid hormones and TSH has been described in the literature; in fact, the usual reverse relationship between the serum levels of TSH and T4 might not be maintained in hypothyroid patients [120]. Patients with severe or long-standing primary hypothyroidism may require three to six months of hormone replacement before the TSH levels are fully suppressed [121], [122], [123]. Conversely, the serum TSH concentration may remain low or normal for up to five weeks after the withdrawal of thyroid hormone replacement when the serum levels of T4 and T3 have already declined to values well below the lower range of normal [120], [123], [124].

Hypothyroidism significantly reduced body weight and elevated the plasma leptin concentration. These findings may be contradictory to the data in human subjects with thyroid disorders, where the majority of the hypothyroid patients suffer from an increased body weight. However, these findings are in agreement with previously reported data on rats [125], [126], [127], [128], [129], [130], [131]; those studies showed that, in hypothyroid rats, the body weight gain per week was very low (approximately one-sixth as high as the weight gain of euthyroid rats) and the body growth was strongly restricted. Additionally, the hypothyroid state was found to increase the serum leptin level; the "satiety hormone" leptin suppresses food intake in hypothyroid rats and may reduce the level of metabolism and body growth gain [132], [133], [134]. Treatment with LT4 decreased the leptin levels and re-established normal body weights.

The association of hypothyroidism with plasma pro-inflammatory markers such as TNF-α and CRP has been demonstrated in some studies [135], [136], [137], [138]. However, a few studies have reported the effect of thyroid hormone therapy on these markers with contradictory results [139], [140]. Our data clearly demonstrate the induction of inflammatory and fibrotic markers in hypothyroid rats; most importantly, this inflammation state was aggravated by the LT4 treatment. Adiponectin levels did not change during hypothyroidism or after LT4 treatment, which is consistent with previous studies [141].

In contrast, hypothyroidism stimulated the secretion of the cardiac stress markers BNP and cTnT in parallel with a global decline in cardiac function along with the development of chamber dilation. The reduced heart weight as well as septum and wall thicknesses in hypothyroid rats seemed contradictory to the cardiomyocyte hypertrophy noticed in histopathology. In fact, hypothyroidism can produce changes that resemble heart failure in many aspects [142], [143]. Indeed, studies have showed that hypothyroidism can lead to chamber dilatation from series addition of sarcomeres despite a reduction in cardiac mass [144], [145], [146], [147]. These cellular changes are recognized as components of heart failure. Furthermore, increased chamber diameter/wall thickness ratio during the decompensated phase of heart failure is reflected by a similar increase in myocyte length/width ratio [145], [148], [149]. This occurs by myocyte lengthening without a change in myocyte cross-sectional area during the transition phase [149], [150]. Similarly, early loss of cardiac mass in rats treated with PTU for 4 weeks was due to a reduction in myocyte cross-sectional area [9], [143]. Apoptosis might be another possible contributor to this cardiac mass loss since T3 treatment has been shown to inhibit cardiomyocyte apoptosis in infarcted myocardium [151]. This cardiac phenotype change was also supported by the up-regulation of hypertrophy markers, myofibroblast differentiation, extracellular matrix components, and pro-fibrotic and pro-inflammatory genes that contribute to tissue stiffness and defects in the structure and contractility of the myocardium. Nonetheless, treatment with LT4 enhanced the global cardiac function and abolished the hypertrophy and extracellular matrix markers. However, deleterious effects of this rapid reversal of hypothyroidism were noticed with the induction of the TGF-β1, IL1 and MCP1 expressions in the myocardium.

Indeed, the cardiovascular effects of hypothyroidism include changes in the expression of both structural and regulatory myocyte genes [6], [20], [152]. Thyroid hormones were shown to have opposite effects on collagen synthesis and the induction of TGF-β1. In fact, thyroid hormones induced this pro-fibrotic marker in cardiomyocyte and fibroblast cultures, whereas they decreased the expression of the collagen I gene. Similarly, cardiac hypertrophy was induced by thyroid hormones without any signs of myocardial fibrosis [40], [50], [153], [154], [155], [156], [157].

Finally, when rapidly reversing hypothyroidism, we observed the development of cardiac injury with a net elevation of the serum cTnT level; leukocyte infiltration was also noted, confirming the presence of myocarditis. This phenomenon may be explained by a direct deleterious effect of the hormone on the heart [89]. Indeed, the induction of the inflammatory IL1 and MCP1 as well as the drastic increases in TGF-β1 might be responsible for this cellular infiltration. TGF-β1 is a multi-potent cytokine that plays an important role in the onset of inflammation [158], [159].

In conclusion, we showed that hypothyroidism is related to the development of cardiac fibrosis and inflammation. Most importantly, the rapid correction of hypothyroidism led to cardiac injuries. This study might offer new insights for the management of hypothyroidism-induced heart disease.

## Supporting Information

Figure S1 **Experimental protocol with different measured parameters.**
(TIF)

Figure S2 **Plasma leptin levels in the different groups of rat treatments at the time of sacrifice.** Data are represented as the mean ± SEM. n = 10 animals for each group. Statistical analysis was performed with Kruskal-Wallis One-way ANOVA on rank tests followed by post hoc Dunn's multiple comparison tests. *p<0.01 *vs.* control and reverse.
(TIF)
